# Abu Dhabi Public Health Workforce Development: Learning Points From the Comparison of Six Countries

**DOI:** 10.3389/phrs.2025.1606334

**Published:** 2025-06-12

**Authors:** Tahani Al Qadiri, Marília Silva Paulo, Mohamud Sheek-Hussein, Katarzyna Czabanowska, Erik Koorneef, Michal Grivna

**Affiliations:** ^1^ Institute of Public Health, College of Medicine and Health Sciences, United Arab Emirates University, Al-Ain, United Arab Emirates; ^2^ NOVA National School of Public Health, Public Health Research Center, Comprehensive Health Research Center, REAL, CCAL, Universidade Nova de Lisboa, Lisbon, Portugal; ^3^ School of PH, Loma Linda University, Loma Linda, CA, United States; ^4^ Department of International Health, Care and Public Health Research Institute, (CAPHRI), Faculty Health, Medicine and Life Sciences, Maastricht University, Maastricht, Netherlands; ^5^ Department of Health Policy and Management, Institute of Public Health, Faculty of Health Sciences, Jagiellonian University, Krakow, Poland; ^6^ Department of PH and Preventive Medicine, Second Faculty of Medicine, Charles University, Prague, Czechia

**Keywords:** public health education, public health workforce development, Abu Dhabi, education of public health professionals, public health professionals

## Abstract

**Objectives:**

This study aimed to review the healthcare systems and the educational public health (PH) the workforce structures in six countries: the United Arab Emirates (UAE), the United States of America (USA), the Kingdom of Saudi Arabia (KSA), the United Kingdom (UK), Canada, and Singapore.

**Methods:**

This review was developed by searching databases from the World Health Organization and the World Bank, official data from each country’s respective ministries of health and National Bureaus of Statistics, the European Public Health Association, and studies conducted by educational institutions.

**Results:**

The USA, the UK, and the KSA showed an insufficient concentration of PH specialists and educational opportunities. In contrast, Singapore and Canada incentivized citizens to pursue PH education, resulting in more PH physicians and specialists. The UAE (Abu Dhabi) was found to remain in its early stages of development.

**Conclusion:**

To strengthen and advance the public health workforce in the UAE (Abu Dhabi) and the countries described, the concept needs to be defined and integrated fully into the entire health system, from academia to the transversal structures of the Ministries of Health.

## Introduction

According to the World Health Organization (WHO), “all persons engaged in activities with the sole purpose of promoting health are regarded as human resources for health” [1]. This definition encompasses those who support the overall effectiveness of a functioning health system, including healthcare workers (HCWs) who care for patients, those who address underlying health factors, and those who focus on the fundamental factors affecting health, as well as others who support the entire endeavor [2]. Public health professionals have been defined as those who are “educated in public health or a related discipline (…) employed to improve health through a population focus.” [3]. The public health workforce includes individuals engaged in public health activities that are the primary part of their role (core public health workforce) [4], and those who contribute to public health activities and essential public health operations as part of their job as well as other professionals whose work may significantly affect population health (wider public health workforce) [4]. Creating Public Health (PH) as a single discipline was a defining moment in the field as it established the discipline, separate and distinct from the practice of medicine. The number of PH schools varies significantly worldwide throughout North America (394), Latin America (124), Asia (448), Europe (457), Oceania (48), and Africa (124), with a global estimated total of over 1597 Schools of PH and/or PH variations [3].

Several factors impacted the competencies of the PH workforce and healthcare professionals during the COVID-19 pandemic and post-pandemic era [4]. These factors prompted further discussion and led to a reformation and redefinition of the capabilities and expertise of the healthcare workforce, and a new model of PH resource administration [4]. In this redefined PH model, public health workers are the ones who understand the situation, contain the spread, minimize the loss of human life, and prevent it from happening again [5]. Recently, the concept of PH has evolved to also include mental health.

The definition of PH was conceptualized by Charles Winslow in 1920 as “the science and practice of extending life, avoiding sickness, and improving health via society’s concerted efforts” [6].

To prevent and control disease in society, PH organizations have been founded to implement regulations and supervise, examine, and monitor the numerous factors that impact PH. These factors include water systems, food manufacturing and processing, sewage disposal, drainage, and contamination.

It is commonly acknowledged that enhancing PH necessitates trans-disciplinary cooperation and requires the collaborative efforts of professionals from various fields and disciplines. This trans-disciplinary cooperation includes healthcare workforce, journalists, pharmacists, social care staff, teachers, retail, and hospitality workers [7]. Nevertheless, many people enter the field of PH through a variety of routes, frequently with specialized knowledge of very limited elements of PH as opposed to a comprehension of the entire scope [8].

The WHO, in cooperation with the Association of Schools of Public Health in the European Regions (ASPHER), launched the WHO-ASPHER Competency Framework for the public health workforce in 2020 to provide guidance for all policymakers, human resources for health, educational institutions, and PH research institutes [9]. In addition to determining, through re-evaluation, the demand for public and global health competencies, this guideline examines the transformative capacity of PH leadership to strengthen health professionals preparedness for global health emergencies [4]. The development of an adequate public health workforce should include in-depth “education, training, development, and evaluation” of the public health workforce to efficiently address priority PH problems and monitor PH activities [4]. Training does not and should not end at the post-secondary and graduate levels. There is a need for continuous in-service training in economics, bioethics, management of human resources, and leadership to attain and enhance the standard of PH service [7]. The certification processes of PH professionals specify the requirements of the workforce appropriate for PH training and experience [7].

The present study aims to contribute to the advancement of the public health workforce in Abu Dhabi, one of the seven Emirates that together form the United Arab Emirates (UAE), by describing its structure across health systems and PH education as developed in the reviewed countries: the United Arab Emirates (UAE), the United States of America (USA), the Kingdom of Saudi Arabia (KSA), the United Kingdom (UK), Canada and Singapore.

## Methods

We conducted a literature review of healthcare systems, the educational structure of PH specialization, and the availability of public health workforce in six countries: the UAE, the USA, the KSA, the UK, Canada, and Singapore. A comprehensive search was done using Google Scholar to get information published by the WHO, the World Bank, ministries of health, and the national bureau of statistics from each country, the European Public Health Association, and international and peer-reviewed studies for each country without time span limitations. Then, the findings were synthesized to interpret the literature and develop a coherent narrative that summarizes the current state of the public health workforce.

## Results

Based on the analysis of the data collected, our results indicated that structural differences exist between the public health workforces in the six countries examined. The results indicate a need for countries to integrate the concept of PH into academic programs and PH competencies for all HCWs to develop a long-term PH strategy.

### Public Health Workforce in the UAE (Abu Dhabi)

**FIGURE 1:**
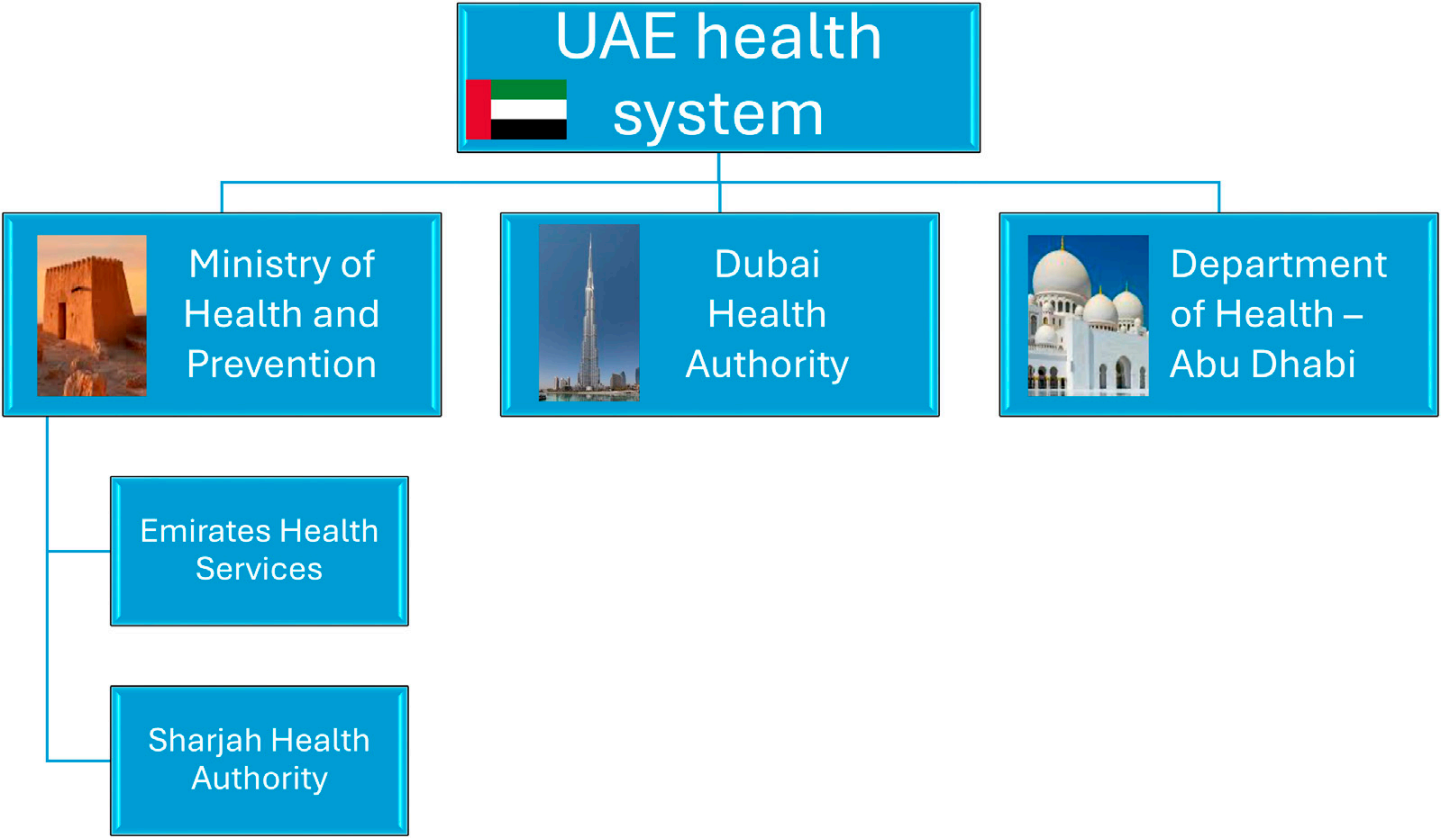
United Arab Emirates health system in 2024.

In the UAE, there is no specific history of PH. The first health facility dates to 1943 in Ras Al Khaimah, followed by another in Al Ain in 1960. As per the records, the first PH concept started to be developed in 1972, when the Ministry of Health creation was announced due to the high mortality rates among the newborn babies [10]. There is limited information about the HCW in the UAE and its qualifications, as the Ministry of Health data indicates only the number of physicians, dentists, pharmacists, technicians, and nurses without their specializations [11]. This is due to the UAE healthcare organization comprising federal and emirates levels (Figure 1). Therefore, this study only focuses on the emirate of Abu Dhabi (excluding the Emirates of Dubai, Ajman, Ras Al-Khaima, Fujairah, Sharjah, Umm Al Quwain).

The Abu Dhabi Healthcare Strategic Plan cements the commitment of the government to advance healthcare services, covering the seven priority areas: (i) reducing capacity gaps, (ii) improving the quality of healthcare services, (iii) patient safety, (iv) retaining qualified healthcare professionals, (v) improving preparedness during emergencies, (vi) ensuring value for money and sustainability of healthcare spending, (vii) introducing an e-health program as a facilitator for the other priorities [12]. Between 2017 and 2019, there was a total of 2483 workers joining the healthcare field annually, totaling 54,231 health professionals registered in the emirate at the end of 2029 if growth remains steady [12]. It is estimated that by 2035, the number of physicians will increase by 50% to cope with the projected population growth and rising rates of chronic diseases. Without significant improvements, rates of obesity, diabetes, and cardiovascular diseases are expected to rise as the juvenile population ages [13, 14]. Cardiovascular diseases (CVD) accounted for 37% of all death cases in 2021, followed by injuries which accounted for 17%, and cancer caused 16%, from which 11.5% were due to breast cancer, 8.7% due to bronchus and lung cancer, and 8.5% due to colon cancer. There is an oversupply of some specializations such as general medicine and gynaecology, while there is an undersupply of physicians specializing in trauma and injury, substance abuse, family medicine, PH, and oncology [12, 13].

### Public Health Education in the UAE

The UAE boasts a highly competitive education industry in the Gulf Cooperation Council (GCC) region, where education is a primary government objective according to the “National Strategy for Higher Education 2030,” a comprehensive strategy devised to provide subsequent cohorts with theoretical and empirical skills to improve the market force [15, 16]. This national strategy also aims to strengthen accreditation standards, set a qualifications framework, and further develop the curricula to match international standards.

A specific professional PH license was introduced in 2011 when the Department of Health in Abu Dhabi (DOH) launched a special PH licensing system initially only targeted at medical doctors [17]. Currently, more than 20 colleges offer PH specializations and degrees varying from bachelor’s to doctorate. The regulatory authorities set the requirements for licensing the graduate colleges per the Unified Healthcare Professional Qualification Requirements (PQR). Recently, in June 2021, the PQR reviewed the requirements for professional qualifications and included new criteria for licensing the PH workforce, such as new titles like PH Medicine, PH Professional, PH-Epidemiology, PH-Biostatistics, and PH-Environmental Health [17]. These recent changes aim to support the UAE healthcare system in establishing a strong PH taskforce.

### Public Health Workforce in the KSA

In 1925, intending to ensure accessibility of healthcare for the visiting (hajj) and reside population, a royal decree launched the first PH authority in Mecca. Later, in 1951, the Saudi Ministry of Health (MOH) was established [18]. The MOH was tasked with the management, supervision, and organization of healthcare and services. The primary task was to provide care and treatment for prevalent diseases and ailments. However, following the declaration of the Alma-Ata in 1978, the policy of the MOH was expanded to include the provision of preventative care with the establishment of primary healthcare [19]. Saudi nationals comprise 44% of the total health workforce and 29.5% of all physicians in the health sector [19]. In nursing, females are significantly underrepresented among Saudi citizens [20]. The health workforce faces significant challenges, including imbalance in professional skills, gender disparities, and accessibility to healthcare challenges. Given the country’s prediction that the senior population would double over the next decade, the KSA is expected to continue spending extensively on healthcare facilities [20]. Life expectancy rises, and so will the need for a competent healthcare workforce over the following decade. The KSA needs a large boost in the number of healthcare providers to fulfill the expanding and aging demands of its population. They would need to employ between 6,000 and 7,000 new nurses every year. The limited training of physicians in the KSA has resulted in a substantial job shortage of family medical practitioners [21]. Around 5% of the doctors in KSA practice family medicine, adding to the country’s healthcare employment challenge and necessitating precise strategic objectives for transformative change [21].

### Public Health Education in the KSA

From 2015 to 2018 there was an increase of 9% in the number of programs offered in PH at both a bachelor and master’s level. In 2018, half of the universities offer bachelor’s degrees in PH or community health, and only six institutions offer master’s degrees in PH [20]. Although certain government and commercial universities in KSA provide PH courses, developing qualified PH practitioners remains challenging. One obstacle is the shortage of a consistent PH curriculum taught in all colleges and educational institutions in the KSA. Since PH is concerned with eradicating and restraining illness, an effective and efficient workforce is needed to lead PH initiatives.

The KSA government is aiming to enhance the healthcare system. “Vision 2030” is a long-term plan for enhancing the country’s public sectors, which include health, education, infrastructure, recreation, and tourism, consequently increasing the country’s economy [20]. Administrative leadership of PH organizations want to limit new recruitment to PH specialties to address a skilled labor shortage in the following areas: epidemiology, environmental health, health education and promotion, food safety, and infection control [20]. A workforce with undergraduate and graduate degrees in PH specialization is necessary sided by the creation of more schools of PH. The introduction of PH courses at both levels, particularly in the discipline of epidemiology, is instrumental in the construction of a competent PH workforce.

### Public Health Workforce in Singapore

Singapore’s aging population raises the demand for chronic care, focusing on pre-existing or long-term sicknesses. Over the previous decade, the number of older persons (over 65) with three or more chronic diseases nearly quadrupled, and by 2030, a quarter of Singapore’s population would be over 65, potentially impacted by chronic illness and the need for chronic care [22]. Increased demand, along with a scarcity of local labor, has resulted in rising healthcare costs—more than Singapore’s GDP growth. In a report published in 2022, the Ministry of Health stressed the importance of health public workers and the need to assist them [22].

According to the Ministry of Health’s Healthcare 2020 Masterplan, an estimated 9,000 health personnel are still needed in PH care. Administrative, executive, and managerial posts in PH/healthcare-related statutory boards and agencies, hospital operations and affairs, healthcare human resource and finance, community health, and regional health systems are available for at least 20% of these (1,800 positions) [23]. To some extent, infection control and workforce planning initiatives influence medical/nursing students’ education, training, and placement.

### Public Health Education in Singapore

The National University of Singapore (NUS), Singapore’s first and only higher education School of PH, has historically offered postgraduate PH education. In 2003, the Institute of Medicine advised that all students should be given access to PH education since it is an important aspect of citizen training [24]. Since 2013, the minor in PH has grown in popularity, with approximately 300 students enrolling annually, prompting NUS to consider offering more advanced PH courses to undergraduates. Concurrent with the worldwide trend of diverse degrees leading to better job opportunities, NUS senior management established second PH majors [24].

During the COVID-19 pandemic, Singapore placed emphasis on residency programs and institutions to address trainees’ anxiety because of the epidemic and the resulting disturbance to their education [25]. According to recent studies, “personnel rosters and work schedules for deployed residents should be” carefully reviewed to ensure students do not spend excessive time away from core tasks essential to their training [25]. The MOH launched job listings for nurses who are not working, working as an agency, or working in non-nursing relevant areas to support the healthcare workforce in a non-COVID capacity. Nurses from non-COVID units were redeployed to care for COVID-19 patients. New deployed nurses required special training, such as managing patient specimens and securing patient transportation. With the requirement for robust infection control measures in healthcare facilities, workflow adjustments requiring precautions and frequent cleaning of equipment/environment were also adopted throughout non-COVID wards [25]. These were invariably followed by employee training.

### Public Health Workforce in Canada

In 2003, after the devastating Severe Acute Respiratory Syndrome (SARS) outbreak, the country’s PH established the federal Public Health Agency of Canada (PHAC) on the following year [26]. Its mission is to promote and sustain the Canadian population’s health, services, and programs and implement regulations designed to enhance health, avoid harm and chronic illnesses, and react to health crises [27]. Front-line providers, consultants, and experts from many fields and professions, such as medicine, nursing, and epidemiology, comprise PH professionals. In Canada, PH services are delivered through a partnership among three levels of government: local, provincial, and federal [27]. While all levels of government share responsibilities for PH, the number and direction of resources allotted for associated activities differ by province and territory. The PH environment in Canada is evolving to suit the country’s rising population’s demands and to address current health concerns, such as adverse health occurrences associated with chronic illnesses and unhealthy lifestyles. Furthermore, it will adapt in response to emerging PH challenges such as introducing tropical diseases, the northward expansion of infectious organisms due to climate change, and disease transmission associated with international travel. PH professionals interact directly with the general public and at-risk populations, or indirectly through monitoring, surveillance, or administrative work.

The percentage of Canadians who do not have access to a family physician or a healthcare team is at an all-time high, producing ripple effects across the health system and impeding patients’ ability to obtain timely care [28]. According to the Canadian Institute for Health Information, the number of physicians in Canada will grow somewhat in 2020 (64). While the statistics are trending in the right direction, they do not fully reflect the challenges related to human health resources, which diminish the overall impact of these improvements. For example, Canada, with 2.7 physicians per 1,000 population, continues to lag behind other Organization for Economic Cooperation and Development member nations, which have 3.5 physicians per 1,000 population [27]. The vast rural areas of Canada present an additional challenge: although 19% of Canadians live in these regions, only 8% of physicians serve them (66). These inadequacies highlight the importance of an all-inclusive Canadian health human resource policy.

### Public Health Education in Canada

The development of PH in Canada dates to 1867 [26]. PH workforce has a variety of educational backgrounds, including training in specific health professions (e.g., medicine, nursing), bachelor’s degrees in PH, and graduate degrees such as research-oriented master’s degrees or professional Master of PH. These professionals work on PH concerns, including occupational health, environmental health, health promotion, infectious illnesses, injuries, and chronic diseases. The PHAC conducts the Canadian Field Epidemiology Program, which offers applied epidemiology training to PH professionals as epidemiologists, PH specialists, doctors, and veterinarians [29]. In addition, the PHAC also provides online professional development courses for professionals. The seven core PH competency categories were formally released after extensive talks with the government, professional organizations, and representatives from numerous disciplines and pilot testing [29]. The epidemic has been identified as the tipping moment for many health personnel already in limited supply.

Before the pandemic, 30% of doctors reported severe levels of stress. COVID-19, conversely, has intensified the spiral of healthcare workforce exhaustion and personnel shortages, putting hospitals at risk. Due to the shortage of primary care capacity, emergency departments are regularly overcrowded, and surgical backlogs continue to grow [30]. Monitoring health workforce supply is crucial for planning future health system demands and responding to disasters like the COVID-19 pandemic. Canada’s two-year family medicine residency training is the quickest of all of the other nations examined, having a three- or four-year program [31].

### Public Health Workforce in the USA

The healthcare system in the USA is divided into three levels: local, state, and federal. With dwindling populations and limited capacity, workforce growth has lagged in PH, posing several issues, including workers’ abilities lagging due to technological advancements, procedures, and data for assessing and monitoring hiring workforce requirements. A society, a multisector health ecosystem, aspires to guarantee that every American has the chance to thrive. The most important job of PH will continue as a custodian of PH, directing the numerous institutions that impact the nation’s health, such as training, infrastructure, and shelter. PH professionals will act as their communities’ primary health strategists, forging cross-sector alliances and defining goals to promote community health while encouraging investment from local companies and others. Robust community markers will be benchmarks for measuring progress toward goals and holding leaders accountable. They simplified this strategic vision into reinforcing characteristics, with the goal of the healthiest nations by building a PH movement, strengthening PH practice, and aligning organizational capacity and infrastructure [32]. In May 2021, the US White House announced plans to invest $7.4 billion to recruit and train public-health workers. During a COVID-19 surge, focusing on anything beyond the immediate pandemic response was hard. However, rebuilding the public health workforce simultaneously is crucial for improving long-term health outcomes. The significant investment in public health comes after the pandemic exposed years of under-investment in the field. For decades, visionary leaders have envisioned a transformed future for public health.

### Public Health Education in the USA

According to data collected in 2020 by the “Council on Education for PH”, in the USA alone, there are 68 Schools of PH and 177 universities that offer a Master of PH or PH-affiliated programs (community medicine, health science, health promotion) [4]. The USA residency model impacts training that includes rotations across multiple sub-specialties in different institutions (with the cessation of cross-institutional rotations), preventing the achievement of target case log numbers and redeployment to outbreak management roles. The COVID-19 pandemic has emphasized the need for a robust and diversified PH workforce, according to the “PH and Social Services Emergency Fund” [33].

### Public Health Workforce in the UK

The UK has a National Health Service (NHS), with local governments granted significant implementation authority. The NHS delivers comprehensive coverage for most health services, including hospitals, general practice, and PH, in three types of care: primary healthcare, secondary care (specialist consultation), and tertiary services. Nowadays, the NHS considers that HCWs pose a more considerable danger to healthcare than financial constraints. Based on current trends, the gap between the number of needed HCWs and the number of available employees might reach around 250,000 by 2030 [34]. If the current trend of HCWs leaving employment continues, and the pipeline of freshly qualified workers and overseas recruits does not grow sufficiently, this figure might climb to more than 350,000 by 2030 [34]. The recent shortages are driven by several factors, including fragmented national responsibility for workforce issues, inadequate career management, reduced financial support for training, stricter immigration policies worsened by Brexit, and an alarmingly high number of physicians and nurses leaving their jobs early. The workforce cannot be addressed without reinvesting in and developing staff. The NHS workforce is likely to remain under strain in the coming years, with insufficient staff facing overwhelming workloads, unrealistic expectations, fatigue, and moral distress, all of which were intensified by the impact of COVID-19.

### Public Health Education in the UK

A survey conducted in 2011 reported the UK had 30 universities offering post-graduate and graduate qualifications in PH [35]. The PH Act of 1848 in the UK set up the General Board of Health, advising on PH issues such as epidemics and illness prevention. It was also given the authority to form and manage local health boards. Tables 1, 2 summarize the overview of the public health workforce under analysis in this review.

**TABLE 1:** Public health approach in the United Arab Emirates, United States of America, Kingdom of Saudi Arabia, United Kingdom, Canada and Singapore up to 2024.

Category	United Arab Emirates	United States of America	Kingdom of Saudi Arabia	United Kingdom	Canada	Singapore
Historical Context	1972, early stages of PH	1872, established system	1925, evolving PH policies	1848, early PH initiatives	1869, evolving PH landscape	2013, recent focus on PH
Public Health Education	Competitive education sector, over 20 colleges offering Public health degrees	68 Schools of PH, extensive MPH programs	Increasing PH programs, lack of standardized curriculum	30 universities offering PH degrees	Diverse educational backgrounds, continuous professional development	NUS offering PH programs, increasing popularity
Workforce Structure	No specific data on specializations, PH license since 2011	Fragmented system, local/state/federal levels	Managed by MOH, high reliance on expatriate workforce	NHS with local government roles	Federal/provincial/local partnership, PHAC established	Ministry of Health managing healthcare, aging population focus
Workforce Statistics	46.19 PH professionals per 10000 (2014), 28.79 doctors per 10000 (2021)	117.13 PH professionals per 10000 (2014), 35.85 doctors per 10000 (2021)	77.74 PH professionals per 10000 (2014), 27.89 doctors per 10000 (2021)	112.5 PH professionals per 10000 (2016), 31.71 doctors per 10000 (2021)	123.8 PH professionals per 10000 (2015), 24.64 doctors per 10000 (2021)	93.97 PH professionals per 10000 (2016), 24.34 doctors per 10000 (2019)
Challenges	Need for structured PH education, licensing	Workforce lagging in technology and procedures, need for investment	Lack of standardized curriculum, insufficient local workforce	Workforce pressure, high attrition rates	Need for continuous adaptation to new health threats	Increased demand for chronic care, rising healthcare costs
Innovations	Abu Dhabi Healthcare Strategic Plan	$7.4 billion investment in PH workforce	Vision 2030 for healthcare improvement	National Health Services long-term workforce plan	Canadian Field Epidemiology Program	Healthcare 2020 Masterplan

**TABLE 2:** Public health overview in the United Arab Emirates, United States of America, Kingdom of Saudi Arabia, United Kingdom, Canada and Singapore up to 2024.

Country	First PH Education Established	PH Recognized as a Clinical Discipline	PH Professionals per Head of Population	Membership in International Organizations	Key messages
UAE	1972	No	46.19 (2014)	None	Early stages, need for structured PH education and licensing
USA	1872	Yes	117.13 (2014)	American Public Health Association, Healthcare without Harm, etc.	Need for robust public health workforce infrastructure, substantial investment in training
KSA	1925	Yes	77.74 (2014)	None	Need for standardized PH curriculum, increasing PH education programs
UK	1848	Yes	112.5 (2016)	Faculty of PH, Royal Society for PH, etc.	High workforce pressure, need for workforce reinvestment
Canada	1869	Yes	123.8 (2015)	CPHA, others	Evolving PH landscape, need for continuous adaptation
Singapore	2013 (for all population as a minor)	Yes	93.97 (2016)	None	Demand for chronic care, need for expanded PH education

Global health Observatory. Data. GHO. Indicators. Medical doctors. 2025 Acessed on 2 Jun 2025 (https://www.who.int/data/gho/data/indicators/indicator-details/GHO/medical-doctors-(per-10-000-population))

## Discussion

Over the years, the vision for PH has centered on building a robust health system that focuses on predicting and proactively preventing illness at the community level rather than providing reactive care once someone becomes sick. Preventing illness can reduce both healthcare costs and the severity of diseases. PH involves the collaborative efforts of many individuals and organizations working to protect and improve the health of populations.

The results indicate a need for countries to integrate the concept of PH into academic programs and PH competencies for all HCWs to develop a long-term PH strategy. WHO has outlined the three fundamental tasks of a PH system: “assessment and monitoring of the health of communities and populations at risk to identify health problems and priorities; formulation of public policies designed to solve identified local and national health problems and priorities; assure that all populations have access to appropriate and cost-effective care, including health promotion and disease prevention services” [36]. These functions are primarily carried out by a “core” PH workforce, such as executives of PH, experts, academics, and practitioners who have been trained in key areas of PH and work in various services in diverse organizations.

The function of a larger group of individuals, PH workforce, in the PH system is becoming more widely recognized. Reliable delivery of public health services depends on effective surveillance and evaluation of population health and wellbeing, assessment of evidence for the efficacy of health and healthcare interventions, utilization of community resources, development and implementation of policies, and strong leadership to ensure the quality of healthcare, preventive services, and overall health-related services. The public health workforce is likely to remain under strain in the coming years if healthcare workers continue to face unreasonable workloads, unrealistic expectations, fatigue, and moral distress, all worsened by the impact of the COVID-19 pandemic. Without sufficient clinical staff, the healthcare system will struggle to recover, let alone thrive. The six countries have a higher number of clinicians per head of population in comparison to the global average/mean (36 per 10.000 population in 2019). However, differences in public health system exist between the six systems/countries [37]. The COVID-19 pandemic has shifted the concept of public health from focusing solely on health promotion, lifestyle, and preventable diseases to a more holistic approach. This new perspective goes beyond traditional clinical health to include reactive care, encompassing social, political, economic, environmental, and geographic determinants. The results indicate a need for countries to integrate the concept of PH into academic programs and PH competencies for all HCWs to develop a long-term PH strategy. Substantially improving health outcomes requires both system transformation and cross-sector collaboration. Today, there are unprecedented opportunities to revamp the fundamental mechanisms of public health, both in terms of public attention and government financial support [38]. There are tremendous challenges: decades-long financial backlog, a worldwide pandemic, ongoing avoidable chronic disease epidemics, the rising threat of climate change, and entrenched injustices that jeopardize our health, longevity, and faith in government and its leadership. A strong PH workforce may positively impact these uncertainties.

In the UAE, the criteria for the PH specialty were not found, and this will certainly negatively affect doctors’ interest in choosing it. In the KSA, despite PH education has shown an increase, it would take a long time to fulfil the need of a competent PH workforce. There are challenges in developing competent PH professionals. One challenge is the lack of a standardized PH curriculum offered in all universities and educational institutions. The administrative leadership of PH organizations is planning to restrict the new recruitment to PH specialty to overcome competent workforce shortage to cover the following specialties: epidemiology, environmental health, PH education and promotion, food safety, and infection control.

In Singapore, the Institute of Medicine recommended that all undergraduates have access to education in PH as it is an essential part of citizen training. By May 2020, volunteers were recruited to form medical teams that went into the dormitories to assist in testing and implementing safe management measures.

The PH landscape in Canada continues to evolve to meet the growing needs of its population and address existing health challenges. Moreover, it continues to adapt further in response to new PH threats. PH professionals are directly involved with the public and subsets of at-risk populations or indirectly involved with the public through monitoring, surveillance, or administrative work.

The UK was one of the first countries in the world to take an early interest in PH. Several critical aspects impact rethinking competency development for PH and other healthcare professionals during COVID-19 and the post-pandemic.

The COVID-19 pandemic has exacerbated existing healthcare challenges, putting immense pressure on overburdened health facilities, increasing surgical backlogs, and pushing healthcare workers to the verge of collapse.

As an example, the main challenges for the public health workforce during the COVID-19 pandemic can be summarized as:

Workforce shortages and high attrition rates: Several countries, such as the USA and the UK, faced a significant shortage of public health professionals, leading to workforce strain. High attrition rates exacerbated these shortages, particularly due to burnout, fatigue, and moral discomfort.

Technological and procedural gap: In countries like the USA, public health professionals struggled with keeping up with technological advancements and updated procedures, which became critical in assessing and monitoring workforce requirements during the pandemic.

Inadequate public health education and training: As observed in countries like Saudi Arabia, gaps existed in the standardized curriculum and continuous professional development. This resulted in a lack of preparedness and sufficient expertise to manage health crises effectively.

Fragmented healthcare systems: Countries with fragmented healthcare systems, such as the USA, faced difficulties coordinating efforts across local, state, and federal levels. This led to inefficiencies in deploying and managing the public health workforce.

Workload and burnout: Public health professionals faced unrealistic workloads, leading to fatigue and burnout, as seen in the UK’s NHS. The pandemic added extra pressure on the already strained workforce, increasing the risk of mental and physical exhaustion.

Need for expanded training and leadership: Continuous in-service training, especially in crisis management, epidemiology, bioethics, and leadership, was identified as crucial to addressing global health emergencies. However, many countries struggled to provide the necessary training, resulting in a lack of preparedness for handling the pandemic effectively.

Chronic disease management: In Singapore and other aging populations, the growing demand for chronic care further strained the public health workforce during the pandemic, as resources were diverted to handle COVID-19 patients.

Insufficient public health infrastructure: Countries like the UAE were still in the early stages of public health workforce development, which limited their ability to manage the pandemic effectively. This highlights the need for structured education and workforce licensing.

These challenges underscored the need for system transformation, enhanced workforce resilience, and cross-sector collaboration to better prepare for future global health emergencies.

A resilient system of health where the focus is to predict and proactively prevent illness at a community level rather than provide reactive care when an individual gets sick. Today, there are unique opportunities both in public attention and government financial support to reform the underlying structures of the PH workforce. However, there are also extreme challenges. PH’s most vital role as a steward of population health will remain unchanged, guiding the disparate systems that influence the nation’s health. PH leaders will serve as principal health strategists of their communities, forming partnerships across sectors and setting goals to improve community health, inspiring investment from local businesses and others.

### Conclusion

This study provides actionable insights and highlights the importance of developing a well-structured and competent public health workforce by comparing these diverse healthcare systems. The analysis underscores the need for universal public health competencies, continuous professional development, and integration of public health into academic and governmental structures. These findings are crucial for Abu Dhabi and other regions aiming to advance their public health systems in preparation for future global health challenges.

### Key Messages

This review provides an overview of PH workforce development, being the first to include countries from the Gulf Cooperation Countries, Middle East.

Innovating PH competencies stretches far beyond individual competence development. Moreover, it is about resilience and preparedness and calls for learning, working, leading, and governing differently.

Critical PH competencies are not limited to the PH workforce but must become relevant for all healthcare professions.

PH must be considered in all areas and at all levels of health workforce education, planning, policy, and governance to achieve transformative capacity toward improved health workforce resilience and preparedness for global PH emergencies.

Evolving disease burden towards a high prevalence of non-communicable diseases, which constitute more than 85% of the disease burden in developed countries.

## Abbreviations

ASPHER, The Association of Schools of PH in the European Region; GCC, Gulf Cooperation Council; KSA, Kingdom of Saudi Arabia; HCWs, Healthcare workers; MOH, Ministry of Health; MPH, Master’s in PH; NUS, National University of Singapore; PHAC, Public Health Agency of Canada; UAE, United Arab Emirates; UK, United Kingdom; USA, United States of America; WHO, World Health Organization.
